# Hospitalizations Associated With Mental Health Conditions Among Adolescents in the US and France During the COVID-19 Pandemic

**DOI:** 10.1001/jamanetworkopen.2022.46548

**Published:** 2022-12-13

**Authors:** Alba Gutiérrez-Sacristán, Arnaud Serret-Larmande, Meghan R. Hutch, Carlos Sáez, Bruce J. Aronow, Surbhi Bhatnagar, Clara-Lea Bonzel, Tianxi Cai, Batsal Devkota, David A. Hanauer, Ne Hooi Will Loh, Yuan Luo, Bertrand Moal, Taha Mohseni Ahooyi, Wanjikũ F. M. Njoroge, Gilbert S. Omenn, L. Nelson Sanchez-Pinto, Andrew M. South, Francesca Sperotto, Amelia L. M. Tan, Deanne M. Taylor, Guillaume Verdy, Shyam Visweswaran, Zongqi Xia, Janet Zahner, Paul Avillach, Florence T. Bourgeois

**Affiliations:** 1 Department of Biomedical Informatics, Harvard Medical School, Boston, Massachusetts; 2 Department of Biostatistics and Biomedical Informatics, Hôpital Saint-Louis, Assistance Publique-Hôpitaux de Paris, Université Paris-Cité, Paris, France; 3 Department of Preventive Medicine, Northwestern University, Chicago, Illinois; 4 Biomedical Data Science Lab, Instituto Universitario de Tecnologías de la Información y Comunicaciones, Universitat Politècnica de València, València, Spain; 5 Department of Biomedical Informatics, Cincinnati Children’s Hospital Medical Center, University of Cincinnati, Cincinnati, Ohio; 6 Department of Pediatrics, Cincinnati Children’s Hospital Medical Center, University of Cincinnati, Cincinnati, Ohio; 7 Department of Biomedical and Health Informatics, The Children’s Hospital of Philadelphia, Philadelphia, Pennsylvania; 8 Department of Learning Health Sciences, University of Michigan Medical School, Ann Arbor; 9 Department of Anaesthesia, National University Health System, Singapore; 10 Unité Informatique et Archivistique Médicale, Bordeaux University Hospital, Bordeaux, France; 11 Department of Psychiatry, University of Pennsylvania Perelman School of Medicine, Philadelphia; 12 Department of Pediatrics (Critical Care), Northwestern University Feinberg School of Medicine, Chicago, Illinois; 13 Department of Pediatrics–Section of Nephrology, Brenner Children’s, Wake Forest University School of Medicine, Winston Salem, North Carolina; 14 Department of Cardiology, Boston Children’s Hospital, Harvard Medical School, Boston, Massachusetts; 15 Department of Biomedical and Health Informatics, The Children’s Hospital of Philadelphia, Philadelphia, Pennsylvania; 16 Department of Pediatrics, University of Pennsylvania Perelman School of Medicine, Philadelphia; 17 Department of Biomedical Informatics, University of Pittsburgh, Pittsburgh, Pennsylvania; 18 Department of Neurology, University of Pittsburgh, Pittsburgh, Pennsylvania; 19 Department of Information Services, Cincinnati Children’s Hospital Medical Center, Cincinnati, Ohio; 20 Department of Biomedical Informatics, Cincinnati Children’s Hospital Medical Center, Cincinnati, Ohio; 21 Department of Pediatrics, Cincinnati Children’s Hospital Medical Center, Cincinnati, Ohio; 22 Computational Health Informatics Program, Boston Children’s Hospital, Boston, Massachusetts; 23 Department of Pediatrics, Harvard Medical School, Boston, Massachusetts

## Abstract

**Importance:**

The COVID-19 pandemic has been associated with an increase in mental health diagnoses among adolescents, though the extent of the increase, particularly for severe cases requiring hospitalization, has not been well characterized. Large-scale federated informatics approaches provide the ability to efficiently and securely query health care data sets to assess and monitor hospitalization patterns for mental health conditions among adolescents.

**Objective:**

To estimate changes in the proportion of hospitalizations associated with mental health conditions among adolescents following onset of the COVID-19 pandemic.

**Design, Setting, and Participants:**

This retrospective, multisite cohort study of adolescents 11 to 17 years of age who were hospitalized with at least 1 mental health condition diagnosis between February 1, 2019, and April 30, 2021, used patient-level data from electronic health records of 8 children’s hospitals in the US and France.

**Main Outcomes and Measures:**

Change in the monthly proportion of mental health condition–associated hospitalizations between the prepandemic (February 1, 2019, to March 31, 2020) and pandemic (April 1, 2020, to April 30, 2021) periods using interrupted time series analysis.

**Results:**

There were 9696 adolescents hospitalized with a mental health condition during the prepandemic period (5966 [61.5%] female) and 11 101 during the pandemic period (7603 [68.5%] female). The mean (SD) age in the prepandemic cohort was 14.6 (1.9) years and in the pandemic cohort, 14.7 (1.8) years. The most prevalent diagnoses during the pandemic were anxiety (6066 [57.4%]), depression (5065 [48.0%]), and suicidality or self-injury (4673 [44.2%]). There was an increase in the proportions of monthly hospitalizations during the pandemic for anxiety (0.55%; 95% CI, 0.26%-0.84%), depression (0.50%; 95% CI, 0.19%-0.79%), and suicidality or self-injury (0.38%; 95% CI, 0.08%-0.68%). There was an estimated 0.60% increase (95% CI, 0.31%-0.89%) overall in the monthly proportion of mental health–associated hospitalizations following onset of the pandemic compared with the prepandemic period.

**Conclusions and Relevance:**

In this cohort study, onset of the COVID-19 pandemic was associated with increased hospitalizations with mental health diagnoses among adolescents. These findings support the need for greater resources within children’s hospitals to care for adolescents with mental health conditions during the pandemic and beyond.

## Key Points

**Question:**

Was the onset of the COVID-19 pandemic associated with changes in the proportion of hospitalizations for mental health conditions among adolescents?

**Findings:**

In this cohort study of 9696 and 11 101 adolescents hospitalized with a mental health condition before and during the pandemic, respectively, at 8 children’s hospitals in the US and France, there was a significant increase in the monthly proportion of hospitalizations associated with mental health conditions following onset of the pandemic.

**Meaning:**

This study showed that global disruption in the first year of the COVID-19 pandemic was associated with increased mental health–related hospitalizations among adolescents.

## Teaser

This cohort study examines changes in the proportion of mental health–associated hospitalizations among adolescents in the US and France during the first year of the COVID-19 pandemic vs before the pandemic.

## Introduction

The COVID-19 pandemic has had substantial effects on the mental health of children and adolescents. Several studies have documented increases in depression, anxiety, and suicidality among adolescents since the start of the pandemic.^1,2,3,4^ This has been attributed, in part, to the extensive disruptions related to social distancing measures, including reductions in routine educational and recreational activities, limited social interactions with peers, and exposure to economic and other stressors in the home.^5,6,7^ Among adolescents, the impact of these factors appears to have been particularly pronounced in females, with greater increases in depressive and anxiety symptoms compared with males.^1,8^

Adolescents with severe mental health conditions may require hospitalization if they are at risk of self-harm and unable to be safely maintained at home. Inpatient psychiatric care for this population is provided in dedicated psychiatric institutions or units tailored to pediatric psychiatric patients. When there are shortages of psychiatric beds, adolescents are frequently boarded in nonpsychiatric units until definitive placement can be provided. Boarding should be minimized as adolescents are typically not able to receive all the mental health services required during this period.^9^ It is unclear whether the proportion of hospitalizations for mental health conditions in pediatric hospitals has changed since the onset of the pandemic. One single-center study of a pediatric hospital in the US found that children and adolescents presenting to the emergency department with mental health conditions during the pandemic were more likely to require admission than in the prepandemic period, pointing to potential increased demand for inpatient mental health services.^10^

Assessing changes in the proportion of hospitalizations for mental health conditions among adolescents is critical to ensure the prioritization of public health programs targeting psychiatric health care needs that have emerged during the pandemic. It is also necessary to better understand the health care infrastructure and resources required to provide high-quality mental health care to these patients. Using data from a multisite cohort of children’s hospitals, we aimed to estimate changes in the monthly proportion of hospitalizations associated with mental health conditions among adolescents following onset of the pandemic.

## Methods

### Study Population

In this cohort study, the study sample was drawn from sites participating in the Consortium for Clinical Characterization of COVID-19 (4CE), an international collaborative focused on facilitating clinical investigations of COVID-19 using federated informatics approaches for the analysis of aggregate and deidentified electronic health record data.^11,12^ All health care systems with a dedicated children’s hospital able to provide data participated in the analysis (eTable 1 in Supplement 1). Approval for the study was obtained from the institutional review board at each site, with a waiver of informed consent because only deidentified patient data were analyzed. The study followed the Strengthening the Reporting of Observational Studies in Epidemiology (STROBE) reporting guideline.

Patients were included in the analysis if they were 11 to 17 years of age and had a hospitalization associated with at least 1 diagnosis of a mental health condition between February 1, 2019, and April 30, 2021. We focused on adolescent patients since mental health conditions are more common in this age group than in children younger than 11 years, and we selected the age range based on standard definitions of adolescence.^13^ Five pediatric hospitals in the US and 3 in France contributed patient-level data to the analyses. The contributing hospitals in the US were Boston Children’s Hospital, Children’s Hospital of Philadelphia, CS Mott Children’s Hospital, Cincinnati Children’s Hospital Medical Center, and Lurie Children’s Hospital of Chicago. The French contributing hospitals were Centre Hospitalier Universitaire de Bordeaux, Hôpital Necker–Enfants Malades, and Hôpital Armand Trousseau. The latter 2 hospitals in France are part of the health care system of Assistance Publique–Hôpitaux de Paris and were analyzed together as a single site.

### Distributed Data Extraction and Analysis Workflow

Data extraction and analyses used a federated approach wherein data were stored and analyzed locally at each site to protect patient confidentiality. Each contributing children’s hospital formatted its data in accordance with a common data standard shared across the 4CE consortium.^11,12^ This initial harmonization step allowed a common script in the R programming language to be run at each site to generate results. The output of locally implemented analysis was shared centrally for additional aggregate analyses. The Structured Query Language scripts used for data harmonization and local analysis at each children’s hospital are publicly available on GitHub,^14^ as is the R code for the intrasite statistical and aggregate analyses.^15^

### Study Periods

To examine changes in the proportion of hospitalizations, we defined 2 periods: a prepandemic period from February 1, 2019, to March 31, 2020 (14 months), and a pandemic period spanning April 1, 2020, to April 30, 2021 (13 months). In keeping with prior studies, we chose April 1, 2020, as the cutoff date to reflect the initiation of mitigation strategies over the latter half of March 2020 following the World Health Organization declaration of the pandemic in mid-March.^10,16^ The end date of April 30, 2021, was selected to capture the first year in which social distancing measures were in place.

### Study Variables and Outcomes

The primary outcome was the change in the monthly proportion of hospitalizations associated with at least 1 mental health condition. Mental health conditions were defined using a modified version of the Child and Adolescent Mental Health Disorders Classification System,^17^ which provides *International Classification of Diseases, Tenth Revision, Clinical Modification* (*ICD-10-CM*) diagnostic codes for 30 mental health disorder groups. Sixteen of these were selected as relevant to adolescent mental health. Modifications were made to add *ICD-10* codes used in France following quality control analysis (the final *ICD-10-CM* and *ICD-10* code list is available on GitHub).^18^ Hospitalizations in which at least 1 of these diagnostic codes was assigned were defined as mental health condition–associated hospitalizations.

Secondary outcomes focused on the change in monthly proportions of hospitalizations for specific mental health condition groups. We included hospitalizations with at least 1 *ICD-10* code for a condition in these condition-specific analyses. Information on patient sex was also collected to allow for stratified analyses.

### Data Quality Control Procedures

As an initial quality control measure, physicians providing patient care during the study period reviewed local summary data to ensure that results were consistent with expected hospitalization patterns for their institution. This included examination of hospitalization counts per month at each health care site (eFigure 1 in Supplement 1). Outliers in the data were identified and investigated and modifications made to the data extraction, as necessary. To identify mental health diagnosis codes that were potentially missing due to differences in the coding systems used for these conditions across health care sites and between countries, we examined the number of *ICD-10* codes per mental health condition used at each health care site (eFigure 2 in Supplement 1). This review revealed systematic differences in the use of *ICD-10* codes between France and the US in suicidality and self-injury, requiring the addition of a specific set of codes used in France.

### Statistical Analysis

To compare the hospitalization characteristics between the 2 periods, we determined the number of unique patients hospitalized in each period and compared patient and hospital characteristics. The χ^2^ test was used to compare categorical variables, the *t* test to compare continuous variables, and quantile regression to compare medians.

To measure the association between pandemic onset and the proportion of hospitalizations, we performed interrupted time-series (ITS) analyses using monthly proportions of hospitalizations for mental health conditions (among all hospitalizations) at each contributing children’s hospital.^19^ Linear regression models were fit to estimate the mean monthly change in this proportion during the 2 studied periods, before and during the pandemic. The ITS analysis represents the difference between the 2 trend lines and is described as the proportion difference (ie, a positive difference indicates an increase in the mean monthly change in hospitalization proportion during the pandemic compared with before the pandemic). We estimated 95% CIs for the proportion differences using parametric bootstrapping (10 000 iterations), assuming each proportion was drawn from a binomial distribution. Significance testing for within-period proportions and proportion differences was performed using *z* test parametric bootstrapped distributions.

The same method was used to examine the change in the proportion of hospitalizations for specific mental health conditions (among all hospitalizations). We focused on the 3 condition groups most prevalent in the study population and also examined eating disorders, which have been reported to have increased during the pandemic in prior studies.^16,20^ Each site then stratified the analyses by sex.

We used random-effects meta-analysis to estimate pooled proportion differences across hospitals and countries. The hospital and country effects were treated as random effects to account for heterogeneity in the effects of the pandemic across populations.

A 2-sided *P* = .05 was used to determine statistical significance. Analyses were performed using R, version 4.0.0 (R Project for Statistical Computing).

## Results

### Study Population

We identified 9696 adolescents who had at least 1 mental health condition–associated hospitalization during the reference prepandemic period and 11 101 during the examined pandemic period. The mean (SD) age at hospitalization of patients in the prepandemic cohort was 14.6 (1.9) years and in the pandemic cohort, 14.7 (1.8) years (Table). There was a greater proportion of females than males hospitalized both during the prepandemic period (5966 [61.5%] vs 3730 [38.5%]; *P* < .001) and during the pandemic (7603 [68.5%] vs 3498 [31.5%]; *P* < .001). The median duration of hospitalization was 7 days both before the pandemic and during the pandemic (IQR, 4-11 days and 4-13 days, respectively).

**Table.** Characteristics of Adolescents Hospitalized With Mental Health Conditions

Characteristic	Adolescents, No. (%)^a^		*P* value^b^
	Prepandemic period (n = 9696)	Pandemic period (n = 11 101)	
Sex			
Female	5966 (61.5)	7603 (68.5)	<.001
Male	3730 (38.5)	3498 (31.5)	
Age at hospital admission, mean (SD), y	14.6 (1.9)	14.7 (1.8)	<.001
Duration of hospitalization, median (IQR), d	7 (4-11)	7 (4-13)	.26
Mental health condition^c^			
Anxiety disorders	5083 (52.4)	6066 (57.4)	<.001
Depressive disorders	4545 (46.9)	5065 (48.0)	.13
Suicidality or self-injury	4101 (42.3)	4673 (44.2)	.005
Mental health symptoms^d^	2133 (22.0)	2532 (24.0)	<.001
Trauma and stressor-related disorders	2105 (21.7)	2352 (22.3)	.34
Eating disorders	1425 (14.7)	1923 (18.2)	<.001
Miscellaneous^e^	1789 (18.5)	1693 (16.0)	<.001
Disruptive, impulse control, and conduct disorders	1601 (16.5)	1591 (15.1)	.005
Substance-related and addictive disorders	745 (7.7)	996 (9.4)	<.001
Somatic symptoms and related disorders	424 (4.4)	457 (4.3)	.90
Schizophrenia spectrum and other psychotic disorders	385 (4.0)	422 (4.0)	.96
Obsessive-compulsive and related disorders	301 (3.1)	419 (4.0)	.001
Bipolar and related disorders	374 (3.9)	394 (3.7)	.66
Sleep-wake disorders	267 (2.8)	335 (3.2)	.09
Personality disorders	270 (2.8)	220 (2.1)	.001
Dissociative disorders	15 (0.2)	19 (0.2)	.86

^a^ All values are based on unique patients hospitalized during each of the 2 periods.

^b^ Statistical tests were χ^2^ tests for hospitalizations, sex, and mental health conditions; *t* tests for age; and quantile regression for median lengths of stay.

^c^ Values represent hospitalization counts. Patients could be diagnosed with more than 1 mental health condition.

^d^ Mental health symptoms included diagnoses such as nervousness, impulsiveness, and emotional lability.^17^

^e^ Miscellaneous symptoms included diagnoses such as neurasthenia, persistent mood disorder, and nonpsychotic mental disorder.^17^

The 3 most prevalent mental health conditions in the study population were anxiety, depression, and suicidality or self-injury during both the prepandemic and the pandemic periods (eFigure 3 in Supplement 1). Adolescents were diagnosed with anxiety disorders in 5083 hospitalizations (52.4%) in the prepandemic period, which increased to 6066 (57.4%) during the pandemic (*P* < .001). The proportion of adolescents diagnosed with suicidality or self-injury also increased significantly between the prepandemic and pandemic periods (4101 [42.3%] to 4673 [44.2%]; *P* = .005), though there was no significant change observed for the proportion of adolescents diagnosed with depression (4545 [46.9%] vs 5065 [48.0%]; *P* = .13).

### Change in Proportion of Hospitalizations

**Figure 1.**
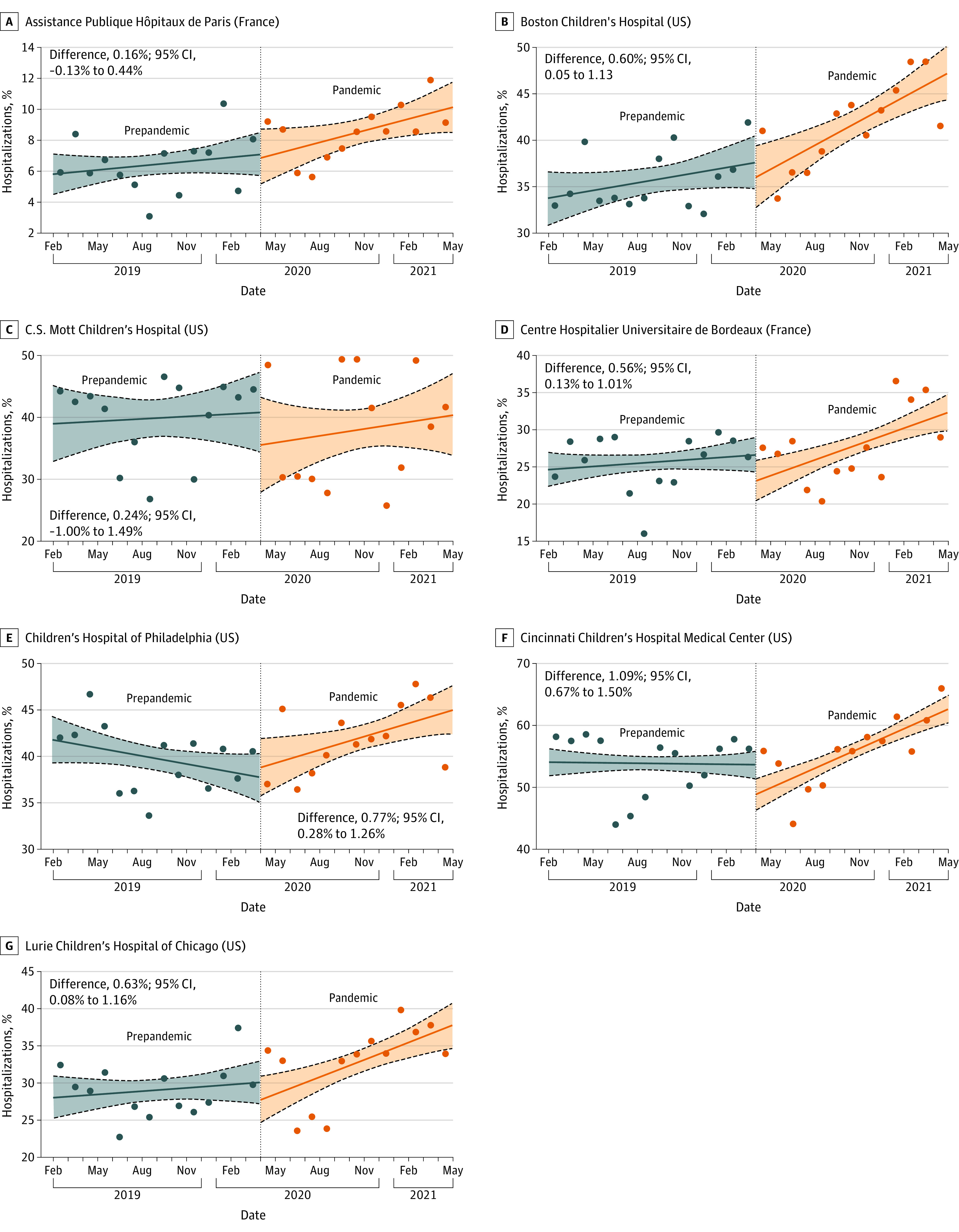
Interrupted Time Series Analysis by Health Care Site Dots represent the proportion of hospitalizations in a given month that included at least 1 mental health diagnosis. Results of the interrupted time series analyses are presented as monthly proportion differences between the prepandemic and pandemic periods. The 95% CIs were estimated using parametric bootstrapping (10 000 iterations). Solid lines represent the medians, the lower and upper dashed lines represent the 2.5th and 97.5th percentiles, respectively, and the shaded area represents the bootstrap iterations between the 2.5th and 97.5th percentiles.

During the prepandemic period, there were a total of 36 059 hospitalizations, of which 12 122 (33.6%) were associated with mental health conditions; during the pandemic, 11 605 of 31 908 hospitalizations (36.4%) were associated with a mental health condition. The monthly proportion of hospitalizations associated with mental health conditions remained stable at each health care site during the prepandemic period (eTable 2 in Supplement 1). In contrast, during the pandemic period, 6 of the 7 sites had significant increases in the monthly proportion of hospitalizations associated with mental health conditions. Compared with the prepandemic period, based on ITS analysis, this represented significant increases in the monthly proportion of adolescents hospitalized with mental health conditions at 4 of the 5 health care sites in the US and 1 of the 2 sites in France (Figure 1).

**Figure 2.**
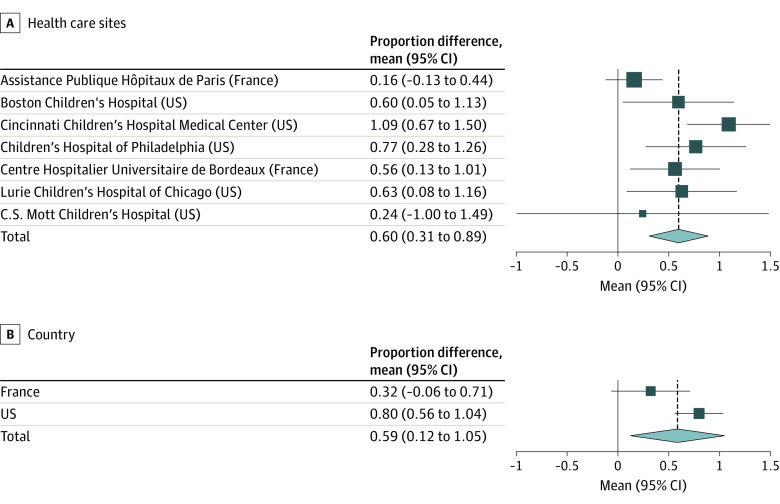
Meta-analysis of Interrupted Time Series Analysis by Health Care Site and Country The proportion difference between the prepandemic and pandemic periods for each health care site or country was determined by interrupted time series analyses. The pooled effect was estimated using a random-effects meta-analysis. Squares represent mean proportion differences, with horizontal lines indicating 95% CIs. The size of the squares is proportional to the relative sample size of each health care site among all sites. Diamonds represent the pooled mean proportion differences among all health care sites, with outer points of the diamonds representing the 95% CIs of the pooled means. The vertical dashed lines represent projections of the pooled mean proportion differences.

Results of the meta-analysis of the ITS at the level of the health care sites are shown in Figure 2. The overall monthly proportion difference was significant, with a mean increase of 0.60% (95% CI, 0.31%-0.89%) during the pandemic period. When aggregated by country, the mean proportion differences were not significant in France (0.32%; 95% CI, −0.06% to 0.71%) but were significant in the US (0.80%; 95% CI, 0.56%-1.04%).

**Figure 3.**
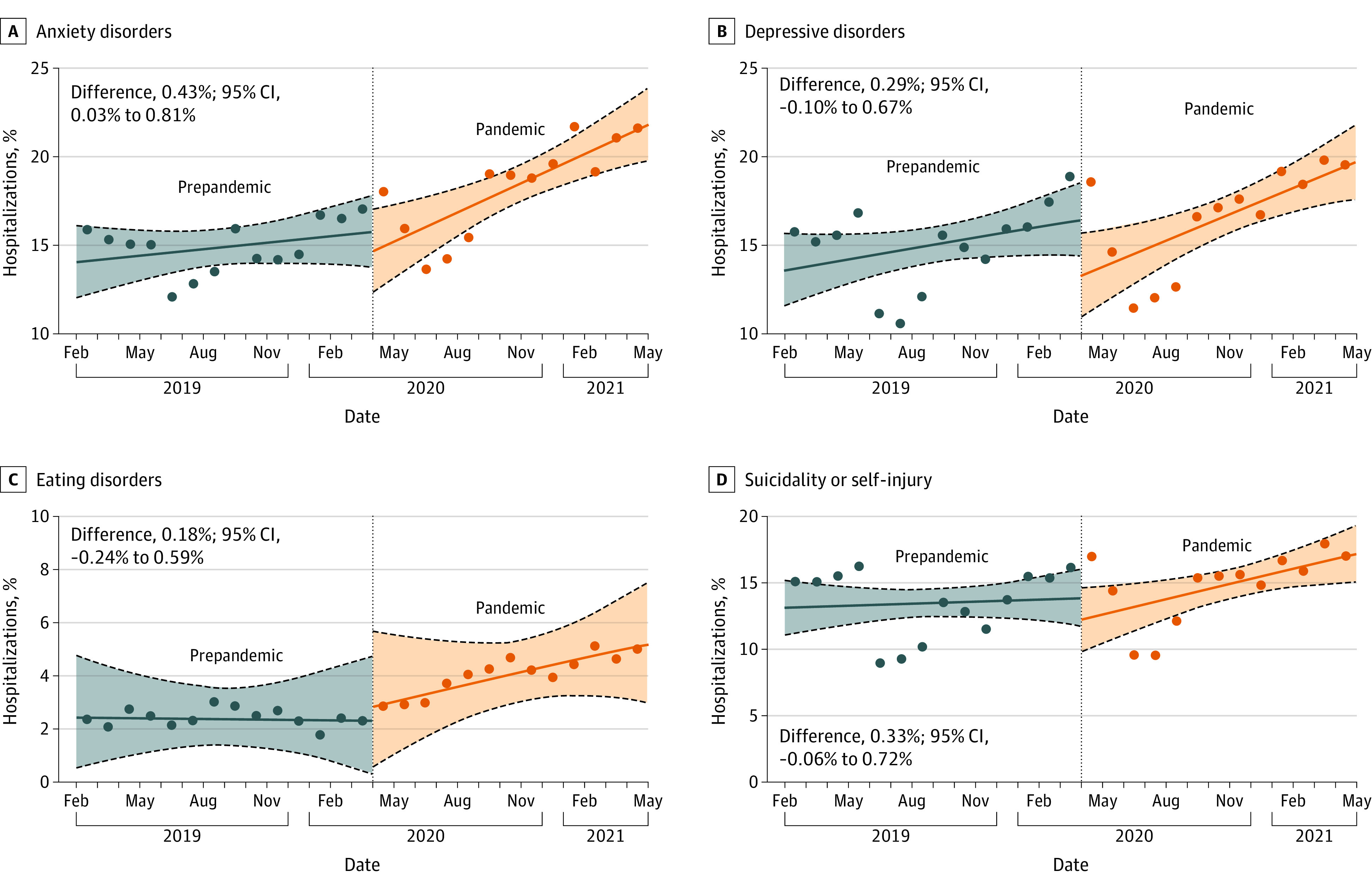
Interrupted Time Series Analysis for Selected Mental Health Conditions Dots represent the proportion of hospitalizations in a given month that included at least 1 of the selected mental health diagnoses. Results of the interrupted time series analyses are presented as monthly proportion differences between the prepandemic and pandemic periods. The 95% CIs were estimated using parametric bootstrapping (10 000 iterations). Solid lines represent the medians, the lower and upper dashed lines represent the 2.5th and 97.5th percentiles, respectively, and the shaded area represents the bootstrap iterations between the 2.5th and 97.5th percentiles.

In the prepandemic period, monthly proportions of hospitalization for anxiety, depression, and suicidality or self-injury were stable. During the pandemic period, there were significant increases in monthly proportions of hospitalizations associated with anxiety disorders (0.55%; 95% CI, 0.26%-0.84%), depressive disorders (0.50%; 95% CI, 0.19%-0.79%), and suicidality or self-injury (0.38%; 95% CI, 0.08%-0.68%). We did not observe a significant increase in hospitalizations for eating disorders (0.18%; 95% CI, −0.16% to 0.49%) (eTable 3 in Supplement 1). For anxiety disorders, this represented a significant increase between the prepandemic and pandemic periods (mean difference, 0.43%; 95% CI, 0.03%-0.81%) (Figure 3).

**Figure 4.**
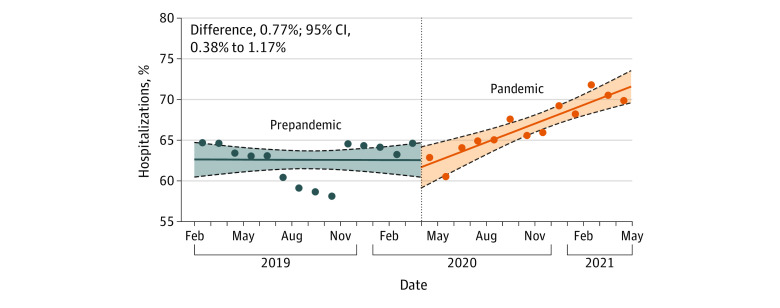
Interrupted Time Series Analysis for Proportion of Hospitalizations Among Females Dots represent the proportion of females among mental health–related hospitalizations across all sites in a given month. The result of the interrupted time series analysis is represented as the monthly proportion difference between the prepandemic and pandemic periods. The 95% CIs were estimated using parametric bootstrapping (10 000 iterations). Solid lines represent the medians, the lower and upper dashed lines represent the 2.5th and 97.5th percentiles, respectively, and the shaded area represents the bootstrap iterations between the 2.5th and 97.5th percentiles.

Figure 4 shows the monthly proportion of hospitalizations for females. During the prepandemic period, there was no increase in monthly proportion of females hospitalized with a mental health condition (0.01%; 95% CI, –0.26% to 0.27%). During the pandemic, there was a significant increase (0.77%; 95% CI, 0.46%-1.07%) (Table). This represented a significant increase between the prepandemic and pandemic periods (mean difference, 0.77%; 95% CI, 0.38%-1.17%).

## Discussion

In this multisite retrospective cohort study, we found that adolescents had a significantly increased proportion of hospitalizations associated with mental health conditions during the first 13 months following onset of the COVID-19 pandemic compared with a 14-month prepandemic period. In this cohort, anxiety, depression, and suicidality or self-injury were the most prevalent conditions, and the proportion of hospitalizations for these conditions increased during the examined pandemic period. Adolescent females had a greater increase in the proportion of hospitalizations associated with mental health conditions during the pandemic compared with males. The temporal increase in mental health condition–associated hospitalization proportions was observed in 4 of the 5 US health care sites and 1 of the 2 health care sites in France, with a between-period proportion difference of 0.60% per month. Our findings extend those of prior studies focusing on the well-being of adolescents during the pandemic^1,2,3,5,6,7,8^ and demonstrate the application of large-scale federated data approaches to increase our understanding of the burden of mental health conditions among adolescents and the rising need for adolescent mental health treatment resources.

Our results are consistent with prior studies, highlighting the potential association of pandemic-related disruptions with adolescent social, emotional, and behavioral well-being.^1,21,22,23,24^ The greater illness burden observed among female adolescents is also in line with findings reported in other analyses.^1,8,24^ These patterns have been documented primarily within individual countries and especially in single-center studies; few robust data exist across multicenter and international settings. Importantly, specific mitigation strategies and timing of containment measures differed across countries throughout the pandemic. Thus, given the scale and global scope of the public health crisis, analyzing overall changes in proportions of hospitalizations across countries provides a measure of the consequences of the pandemic for adolescents that is specific to neither individual mitigation measures nor national settings. Quantifying the change in mental health burden provides data to guide future policies and public health programs globally to improve access to high-quality mental health care in the outpatient and inpatient settings, even after the COVID-19 pandemic. In the short term, these findings support efforts to improve screening and detection of several mental health conditions and their related complications and to inform programs seeking to ensure widespread availability of mental health access, including through telemedicine, school-based programs, and community mental health services and response teams.^25^

It is important to note that prior to the onset of the COVID-19 pandemic, more than 40% of hospitalized adolescents in this study’s cohort had a diagnosis of anxiety, depression, or suicidality or self-injury. This is consistent with the high mental health burden observed among children and adolescents before 2020.^26,27,28^ Since 2010, rates of suicide have been rising in this population, and suicide represented the third leading cause of death among individuals 10 to 24 years of age in 2020.^29^ While the factors contributing to these conditions are not fully known, the social isolation and other disruptions related to the pandemic may have exacerbated underlying trends and led to the recent declaration by the American Academy of Pediatrics of a national emergency in child and adolescent mental health.^25^ This declaration was accompanied by a set of recommendations for strategies to support improved mental health delivery to this population, including approaches to reduce patient boarding in nonpsychiatric units and increase access to acute psychiatric treatment.

The 4CE network leverages a multisite research infrastructure based on routinely collected health care data to perform COVID-19–related clinical analyses expeditiously and at scale while preserving patient confidentiality.^11^ While the focus to date has been on COVID-19, the contemporaneous efforts on methods development, ongoing addition of new hospital sites across health care systems, and open-source release of all code has produced a robust infrastructure that supports ongoing collaborative research studies on diverse topics.^30,31,32^ In particular, the 4CE infrastructure will be able to support continued monitoring of health care utilization for mental health conditions among adolescents, including assessing trends in specific conditions and identifying patient groups at greatest risk for mental health disorders.

### Limitations

There are several limitations to this study. First, we were unable to ascertain the primary reason for hospitalization among adolescents with a mental health condition and instead identified all hospitalizations associated with at least 1 mental health diagnosis to examine trends over time. This is a known limitation with the use of diagnostic codes, and ongoing 4CE efforts are focused on distinguishing specific admission diagnoses vs incidental ones.^33^ Second, variables selected for structured data extraction across sites were prespecified by the 4CE network, limiting our ability to explore additional hospital and patient characteristics. This is a trade-off for efficiently implementing multiple simultaneous analyses across the 4CE network. Third, our modeling relied on the assumption of linearity of underlying temporal trends. In particular, we did not account for seasonality, as this would have required fitting a model over several consecutive years, and we only had 1 year of data in the prepandemic period.^34,35^ However, such adjustment may not be required, as findings from studies examining the association between seasons and affective disorders remain inconclusive.^36^ In addition, the use of proportions rather than absolute counts served to mitigate potential patterns in overall hospital admission.

## Conclusions

This large, multisite cohort study provides estimates of the increased proportion of adolescent hospitalizations associated with mental health conditions during the COVID-19 pandemic. Based on data from 8 children’s hospitals across 2 countries, we observed high proportions of hospitalizations associated with anxiety, depression, and suicidality or self-injury during the pandemic. The increase in hospitalizations was particularly notable for adolescent females. These findings support the need for ongoing prioritization of resources for children’s hospitals to provide care to adolescents with mental health conditions.

## Notes

Supplement 1. **eTable 1.** Contributing Children’s Hospitals **eTable 2.** Changes in the Monthly Proportion of Hospitalizations Across Health Care Sites Between the Reference Prepandemic Period and the Examined Pandemic Period **eTable 3.** Changes in the Monthly Proportion of Hospitalizations for Selected Mental Health Conditions Across Health Care Sites Between the Reference Prepandemic Period and the Examined Pandemic Period **eFigure 1.** Number of Hospitalizations per Month **eFigure 2.** Use of *ICD-10* Codes by Health Care Site **eFigure 3.** Counts of Patients per Month Stratified by Mental Health Condition

Supplement 2.
The Consortium for Clinical Characterization of COVID-19 by EHR (4CE)

Supplement 3.
Data Sharing Statement
