# Supplementary material for: Hospitalizations Associated With Mental Health Conditions Among Adolescents in the US and France During the COVID-19 Pandemic
Source: JAMA Netw Open. 2022 Dec 13;5(12):e2246548. doi: 10.1001/jamanetworkopen.2022.46548 (PMC9856226; doi:10.1001/jamanetworkopen.2022.46548)
Supplement: Supplement 1. — eTable 1. Contributing Children’s Hospitals eTable 2. Changes in the Monthly Proportion of Hospitalizations Across Health Care Sites Between the Reference Prepandemic Period and the Examined Pandemic Period eTable 3. Changes in the Monthly Proportion of Hospitalizations for Selected Mental Health Conditions Across Health Care Sites Between the Reference Prepandemic Period and the Examined Pandemic Period eFigure 1. Number of Hospitalizations per Month eFigure 2. Use of ICD-10 Codes by Health Care Site eFigure 3. Counts of Patients per Month Stratified by Mental Health Condition [file jamanetwopen-e2246548-s001.pdf]

Supplementary Online Content

Gutiérrez-Sacristán A, Serret-Larmande A, Hutch MR, et al; Consortium for Clinical Characterization of COVID-19 by EHR (4CE). Hospitalizations associated with mental health conditions among adolescents in the US and France during the COVID-19 pandemic. *JAMA Netw Open*. 2022;5(12):e2246548. doi:10.1001/jamanetworkopen.2022.46548

eTable 1. Contributing Children's Hospitals
eTable 2. Changes in the Monthly Proportion of Hospitalizations Across Health Care Sites Between the Reference Prepandemic Period and the Examined Pandemic Period
eTable 3. Changes in the Monthly Proportion of Hospitalizations for Selected Mental Health Conditions Across Health Care Sites Between the Reference Prepandemic Period and the Examined Pandemic Period
eFigure 1. Number of Hospitalizations per Month
eFigure 2. Use of *ICD-10* Codes by Health Care Site
eFigure 3. Counts of Patients per Month Stratified by Mental Health Condition

This supplementary material has been provided by the authors to give readers additional information about their work.

© 2022 Gutiérrez-Sacristán A et al. *JAMA Network Open*.

eTable 1. Contributing Children's Hospitals

Country	Children's Hospitals	City	Hospitals per healthcare site	Pediatric beds	Pediatric hospitalizations per year	Obfuscation threshold for small counts[1]
France	Assistance Publique - Hôpitaux de Paris : Hôpital Necker-Enfants Malades and Hôpital Armand Trousseau	Paris	2	443; 163	18,069; 9,686	none
	Centre Hospitalier Universitaire de Bordeaux	Bordeaux	1	280	20,889	3
USA	Boston Children's Hospital	Boston, MA	1	404	28,000	none
	The Children's Hospital of Philadelphia	Philadelphia, PA	1	591	25,003	none
	Cincinnati Children's Hospital Medical Center	Cincinnati, OH	1	624	17,400	none
	C.S. Mott Children's Hospital	Ann Arbor, MI	1	346	7,814	none
	Lurie Children's Hospital of Chicago	Chicago, IL	1	360	12,000	none

[1] This threshold refers to the minimum patient count that will be reported by a hospital for any variables or outcomes in order to ensure patient privacy.

© 2022 Gutiérrez-Sacristán A et al. *JAMA Network Open*.

eTable 2. Changes in the Monthly Proportion of Hospitalizations Across Health Care Sites Between the Reference Prepandemic Period and the Examined Pandemic Period

Healthcare Site	Mean change in monthly porportion of hospitalizations (95% CI)		ITS analysis: difference between time periods (95% CI)
	Pre-pandemic period	During pandemic period	
Assistance Publique - Hôpitaux de Paris	0.09% (-0.08%–0.26%)	0.25% (0.02%–0.47%)	0.16% (-0.13%–0.44%)
Centre Hospitalier Universitaire de Bordeaux	0.14% (-0.13%–0.42%)	0.70% (0.36%–1.04%)	0.56% (0.13%–1.01%)
Boston Children’s Hospital	0.27% (-0.08%–0.62%)	0.87% (0.45%–1.28%)	0.60% (0.05%–1.13%)
Cincinnati Children's Hospital Medical Center	-0.03% (-0.29%–0.23%)	1.06% (0.75%–1.38%)	1.09% (0.67%–1.50%)
Lurie Children's Hospital of Chicago	0.14% (-0.21%–0.49%)	0.77% (0.34%–1.18%)	0.63% (0.08%–1.16%)
Children’s Hospital of Philadelphia	-0.29% (-0.6%–0.02%)	0.48% (0.09%–0.86%)	0.77% (0.28%–1.26%)
C.S. Mott Children’s Hospital	0.13% (-0.64%–0.92%)	0.38% (-0.59%–1.34%)	0.24% (-1%–1.49%)

© 2022 Gutiérrez-Sacristán A et al. *JAMA Network Open*.

eTable 3. Changes in the Monthly Proportion of Hospitalizations for Selected Mental Health Conditions Across Health Care Sites Between the Reference Prepandemic Period and the Examined Pandemic Period

Condition	Mean change in monthly proportion of hospitalizations (95% CI)		ITS analysis: difference between time periods (95% CI)
	Prepandemic period	During pandemic period	
Anxiety disorders	0.12% (-0.13%–0.37%)	0.55% (0.26%–0.84%)	0.43% (0.03%–0.81%)
Depressive disorders	0.21% (-0.05%–0.45%)	0.50% (0.19%–0.79%)	0.29% (-0.1%–0.67%)
Suicidality or self-injury	0.05% (-0.2%–0.31%)	0.38% (0.08%–0.68%)	0.33% (-0.06%–0.72%)
Eating disorders	-0.01% (-0.28%–0.26%)	0.18% (-0.16%–0.49%)	0.18% (-0.24%–0.59%)

© 2022 Gutiérrez-Sacristán A et al. *JAMA Network Open*.

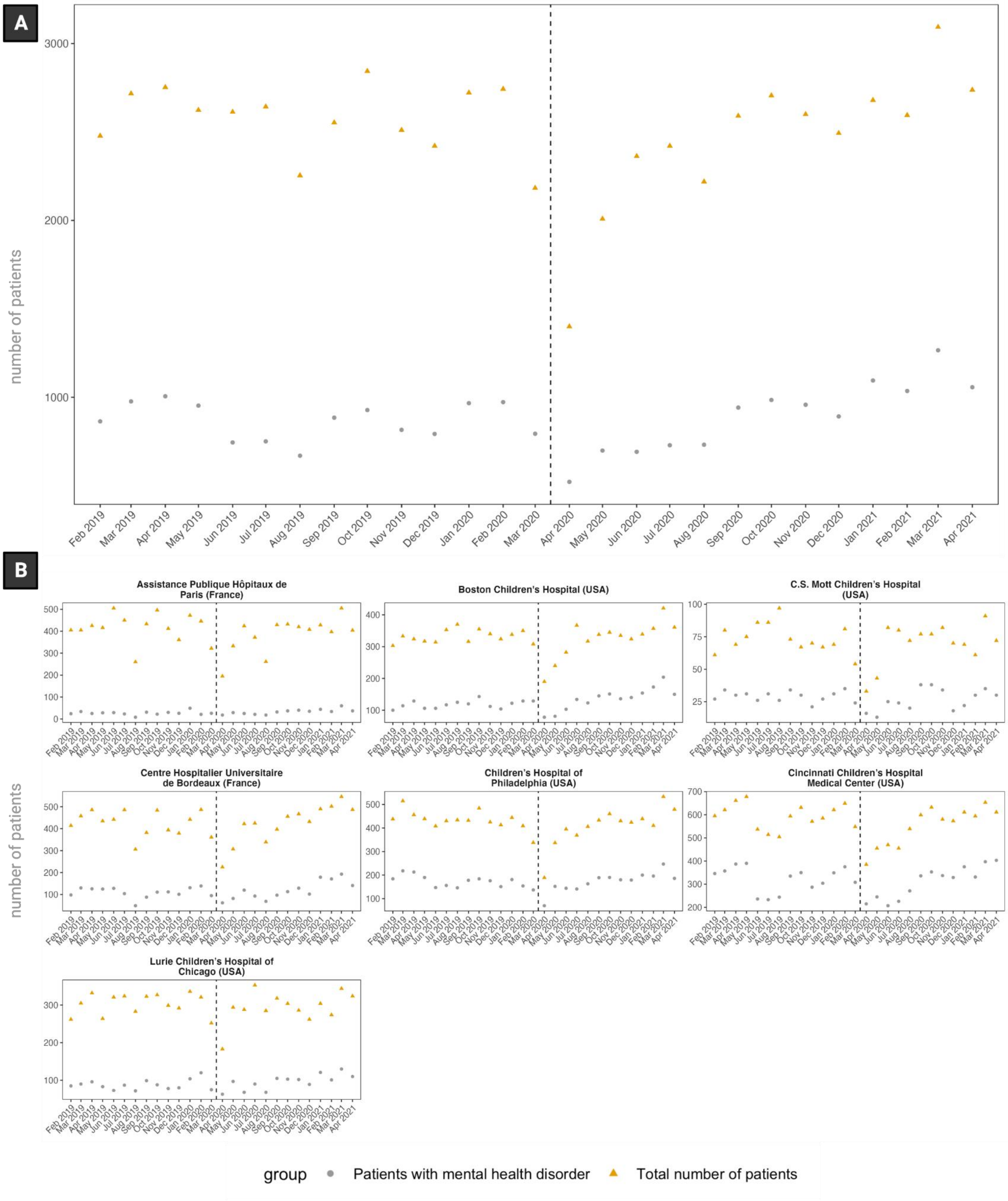

eFigure 1. **Number of hospitalizations per month.** Panel A shows the overall number of monthly hospitalizations with mental health conditions. Panel B shows the number of monthly hospitalizations with mental health conditions per contributing healthcare site. Data extracted from each site was systematically evaluated for irregular patterns in the total number of hospitalizations and number of hospitalizations associated with mental health conditions.

© 2022 Gutiérrez-Sacristán A et al. *JAMA Network Open*.

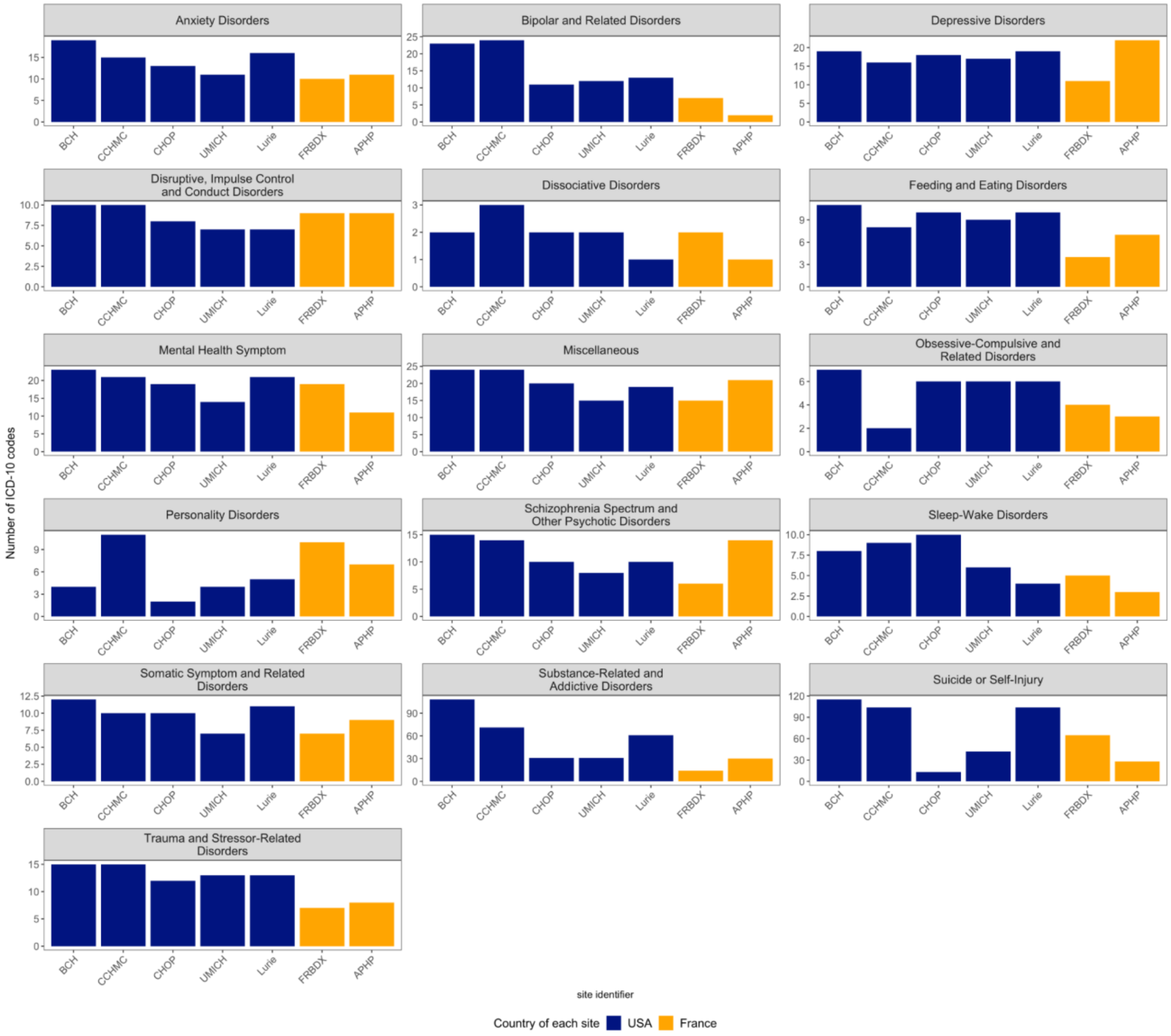

eFigure 2. **Use of *ICD-10* codes by health care site.** Each panel shows the number of ICD-10 codes used by healthcare sites for each of the mental health condition groups. The use of specific ICD-10 codes was examined across healthcare sites and between countries to ensure consistent use of codes for the conditions of interest. This review revealed systematic differences in the use of ICD-10 codes between France and the US, mostly for suicidality and self-injury, requiring addition of a specific set of codes. After adjustment, the final number of ICD-10 codes entailed 1785 codes, among which 1449 were from the original Child and Adolescent Mental Health Disorders Classification System, and 336 were added to reflect French coding practice.

© 2022 Gutiérrez-Sacristán A et al. *JAMA Network Open*.

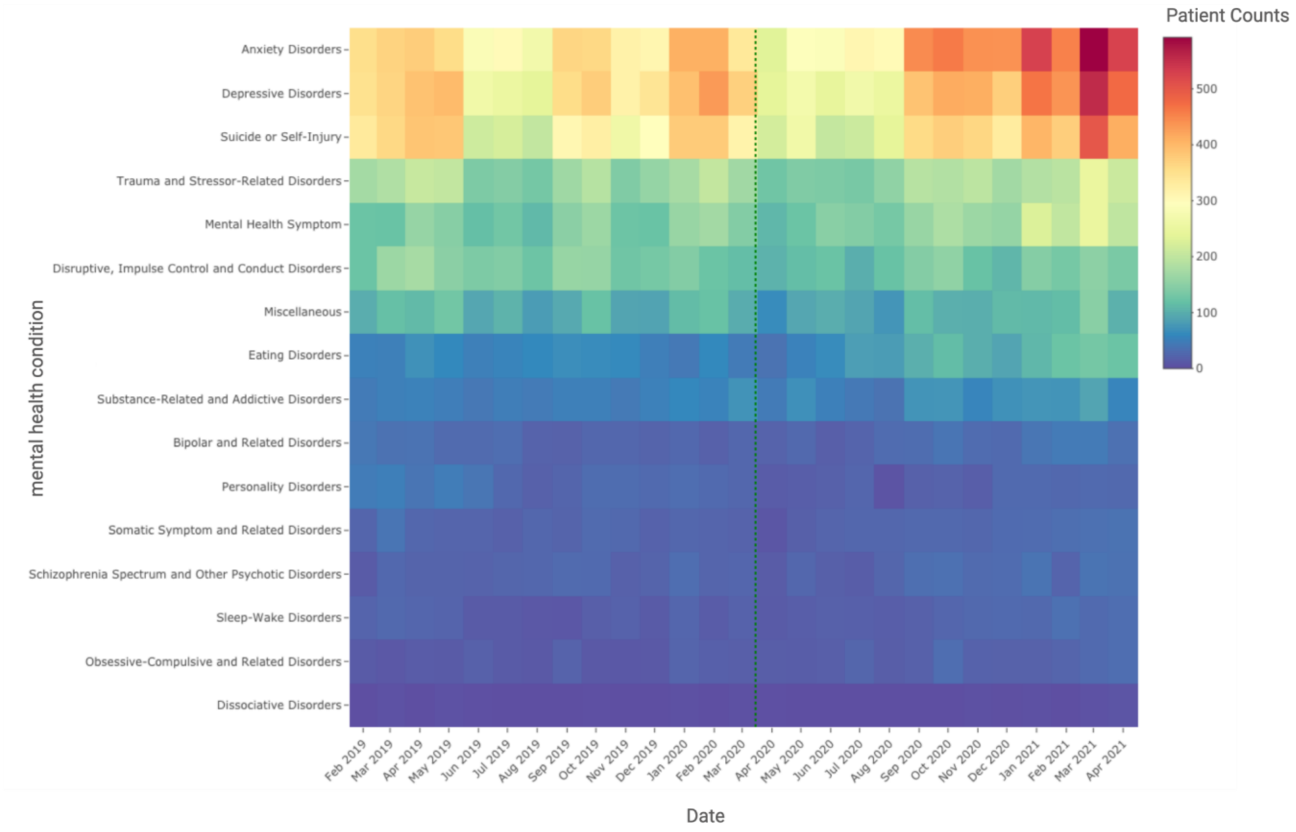

eFigure 3. **Counts of patients per month stratified by mental health condition.** The color of each cell represents the number of patients diagnosed with a given condition across the participating healthcare sites each month. The heatmap is sorted by frequency, from most to least common mental health condition groups.

© 2022 Gutiérrez-Sacristán A et al. *JAMA Network Open*.
