# Supplementary material for: Hospitalizations Associated With Mental Health Conditions Among Adolescents in the US and France During the COVID-19 Pandemic
Source: JAMA Netw Open. 2022 Dec 13;5(12):e2246548. doi: 10.1001/jamanetworkopen.2022.46548 (PMC9856226; doi:10.1001/jamanetworkopen.2022.46548)
Supplement: Supplement 2. — The Consortium for Clinical Characterization of COVID-19 by EHR (4CE) [file jamanetwopen-e2246548-s002.pdf]

Supplemental Online Content: Nonauthor Collaborators
*First name, last name, and suffix (if applicable) are required and will appear in PubMed.

***Group Name(s): The Consortium for Clinical Characterization of COVID-19 by EHR (4CE)**

***First Name and Middle Initial(s)**	***Last Name**	***Suffix (eg, Jr, III)**	Academic Degrees	Institution	Location (city, state/province, country)	Role or Contribution, eg, chair, principal investigator	Group (if more than 1 Group listed in the byline) and/or Subgroup (eg, Steering Committee)
James R	Aaron		MHA	University of Kentucky	Lexington, KY, United State	Member	
Giuseppe	Agapito		PhD	University Magna Graecia of Catanza	Catanzaro, Italy	Member	
Adem	Albayrak			Health Catalyst, INC.	Cambridge, MA, United Sta	Member	
Giuseppe	Albi		MS	University of Pavia, Italy	Pavia, Italy	Member	
Mario	Alessiani		MD, FACS	ASST Pavia, Lombardia Region Heal	Pavia, Italy	Member	
Anna	Alloni		PhD	BIOMERIS (BIOMedical Research Inf	Pavia, Italy	Member	
Danilo F	Amendola		MSc	Clinical Research Unit of Botucatu Me	Botucatu, Brazil	Member	
François	Angoulvant		MD,PhD	Hôpital Necker-Enfants Malades, Ass	Paris, France	Member	
Li L.L.J	Anthony			National Center for Infectious Diseas	Singapore, Singapore	Member	
Fatima	Ashraf		MS	The University of Texas Health Scien	Houston, TX, United States	Member	
Andrew	Atz		MD	Medical University of South Carolina	Charleston, SC, United Stat	Member	
Paula S	Azevedo		MD, PhD	Botucatu Medical School, São Paulo	Botucatu, Brazil	Member	
James	Balshi			St. Luke's University Health Network,	Bethlehem, PA, United Stat	Member	
Brett K	Beaulieu-Jones		PhD	Harvard Medical School	Boston, MA, United States	Member	
Douglas S	Bell			David Geffen School of Medicine at U	Los Angeles, CA, United Sta	Member	
Antonio	Bellasi		MD, PhD	Ente Ospedaliero Cantonale, Lugano	Lugano, Switzerland	Member	
Riccardo	Bellazzi		MS, PhD	University of Pavia, Italy	Pavia, Italy	Member	
Vincent	Benoit		PhD	APHP Greater Paris University Hospi	Paris, France	Member	
Michele	Beraghi		MS	ASST Pavia	Voghera, Italy	Member	
José Luis	Bernal-Sobrino		MS	Hospital Universitario 12 de Octubre,	Madrid, Spain	Member	
Mélodie	Bernaux			APHP Greater Paris University Hospi	Paris, France	Member	
Romain	Bey			APHP Greater Paris University Hospi	Paris, France	Member	
Alvar	Blanco-Martínez		MS	Hospital Universitario 12 de Octubre,	Madrid, Spain	Member	
Martin	Boeker			Technical University of Munich	Munich, Germany	Member	
John	Booth		MSc	Great Ormond Street Hospital for Chi	London, United Kingdom	Member	
Silvano	Bosari		Prof.	IRCCS Ca' Granda Ospedale Maggio	Milan, Italy	Member	
Robert L	Bradford			North Carolina Translational and Clin	Chapel Hill, NC, United Stat	Member	
Gabriel A	Brat		MD	Harvard Medical School	Boston, MA, United States	Member	
Stéphane	Bréant			APHP Greater Paris University Hospi	Paris, France	Member	

Supplemental Online Content: Nonauthor Collaborators
*First name, last name, and suffix (if applicable) are required and will appear in PubMed.

***First Name and Middle Initial(s)**	***Last Name**	***Suffix (eg, Jr, III)**	Academic Degrees	Institution	Location (city, state/province, country)	Role or Contribution, eg, chair, principal investigator	Group (if more than 1 Group listed in the byline) and/or Subgroup (eg, Steering Committee)
Nicholas W	Brown		MEng	Harvard Medical School	Boston, MA, United States	Member	
Raffaele	Bruno		MD	Fondazione I.R.C.C.S. Policlinico Sar	Pavia, Italy	Member	
William A	Bryant		PhD	Great Ormond Street Hospital for Chi	London, United Kingdom	Member	
Mauro	Bucalo		MS	BIOMERIS (BIOMedical Research Inf	Pavia, Italy	Member	
Emily	Bucholz		MD, PhD, N	Boston Children's Hospital, Harvard N	Boston, MA, United States	Member	
Anita	Burgun			APHP Greater Paris University Hospi	Paris, France	Member	
Mario	Cannataro		M.Sc.	University Magna Graecia of Catanza	Catanzaro, Italy	Member	
Aldo	Carmona			St. Luke's University Health Network,	Bethlehem, PA, United Stat	Member	
Charlotte	Caucheteux			Université Paris-Saclay, Inria, CEA	Palaiseau, France	Member	
Julien	Champ			INRIA Sophia-Antipolis – ZENITH tea	Montpellier, France	Member	
Jin	Chen		PhD	University of Kentucky	Lexington, KY, United State	Member	
Krista Y	Chen		BS	Boston Children's Hospital	Boston, MA, United States	Member	
Luca	Chiovato		MD, PhD	Istituti Clinici Scientifici Maugeri SpA	Pavia, Italy	Member	
Lorenzo	Chiudinelli		PhD	ASST Papa Giovanni XXIII, Bergamo	Bergamo, Italy	Member	
Kelly	Cho		PhD, MPH	VA Boston Healthcare System	Boston, MA, United States	Member	
James J	Cimino		MD	University of Alabama at Birmingham	Birmingham, AL, United Sta	Member	
Tiago K	Colicchio		PhD, MBA	University of Alabama at Birmingham	Birmingham, AL, United Sta	Member	
Sylvie	Cormont			APHP Greater Paris University Hospi	Paris, France	Member	
Sébastien	Cossin			Bordeaux University Hospital / ERIAS	Bordeaux, France	Member	
Jean B	Craig		PhD	Medical University of South Carolina	Charleston, SC, United Stat	Member	
Juan Luis	Cruz-Bermúdez		PhD	Hospital Universitario 12 de Octubre,	Madrid, Spain	Member	
Jaime	Cruz-Rojo		MD	Hospital Universitario 12 de Octubre,	Madrid, Spain	Member	
Arianna	Dagliati		MS, PhD	University of Pavia, Italy	Pavia, Italy	Member	
Mohamad	Daniar		MSIS	Boston Children's Hospital	Boston, MA, United States	Member	
Christel	Daniel			APHP Greater Paris University Hospi	Paris, France	Member	
Priyam	Das		PhD	Harvard Medical School	Boston, MA, United States	Member	
Audrey	Dionne		MD	Boston Children's Hospital, Harvard N	Boston, MA, United States	Member	
Rui	Duan		PhD	Harvard T.H. Chan School of Public H	Boston, MA, United States	Member	
Julien	Dubiel			APHP Greater Paris University Hospi	Paris, France	Member	
Scott L	DuVall		PhD	VA Salt Lake City Health Care Syster	Salt Lake City, United State	Member	
Loic	Esteve			SED/SIERRA, Inria Centre de Paris	Paris, France	Member	

*First name, last name, and suffix (if applicable) are required and will appear in PubMed.

***First Name and Middle Initial(s)**	***Last Name**	***Suffix (eg, Jr, III)**	Academic Degrees	Institution	Location (city, state/province, country)	Role or Contribution, eg, chair, principal investigator	Group (if more than 1 Group listed in the byline) and/or Subgroup (eg, Steering Committee)
Hossein	Estiri		PhD	Massachusetts General Hospital	Boston, MA, United States	Member	
Shirley	Fan			University of Michigan	Ann Arbor, MI, United Stat	Member	
Robert W	Follett		BS	David Geffen School of Medicine at U	Los Angeles, CA, United Sta	Member	
Thomas	Ganslandt		MD	University Medicine Mannheim, Heide	Mannheim, Germany	Member	
Noelia	García-Barrio		MS	Hospital Universitario 12 de Octubre,	Madrid, Spain	Member	
Lana X	Garmire		PhD	University of Michigan	Ann Arbor, MI, United Stat	Member	
Nils	Gehlenborg			Harvard Medical School	Boston, MA, United States	Member	
Emily J	Getzen		MS	Perelman School of Medicine at the U	Philadelphia, PA, United St	Member	
Alon	Geva		MD, MPH	Boston Children's Hospital	Boston, MA, United States	Member	
Tomás	González González		MD	Hospital Universitario 12 de Octubre,	Madrid, Spain	Member	
Tobias	Gradinger		MD, BSc	University Medicine Mannheim, Heide	Mannheim, Germany	Member	
Alexandre	Gramfort			Université Paris-Saclay, Inria, CEA	Palaiseau, France	Member	
Romain	Griffier			Bordeaux University Hospital / ERIAS	Bordeaux, France	Member	
Nicolas	Griffon			APHP Greater Paris University Hospi	Paris, France	Member	
Olivier	Grisel			Université Paris-Saclay, Inria, CEA	Palaiseau, France	Member	
Pietro H	Guzzi		PhD	university of catanzaro department of	Catanzaro, Italy	Member	
Larry	Han		PhD	Harvard T.H. Chan School of Public H	Boston, MA, United States	Member	
Christian	Haverkamp		MD	Faculty of Medicine and Medical Cen	Freiburg, Germany	Member	
Derek Y	Hazard		MSc	Institute of Medical Biometry and Stat	Freiburg, Germany	Member	
Bing	He		PhD	University of Michigan	Ann Arbor, MI, United Stat	Member	
Darren W	Henderson		BS	University of Kentucky	Lexington, KY, United State	Member	
Martin	Hilka			APHP Greater Paris University Hospi	Paris, France	Member	
Yuk-Lam	Ho		MPH	VA Boston Healthcare System	Boston, MA, United States	Member	
John H	Holmes		MS, PhD	University of Pennsylvania Perelman	Philadelphia, PA, United St	Member	
Chuan	Hong		PhD	Duke University and Harvard Medical	Durham, NC, United States	Member	
Kenneth M	Huling		HS	Harvard Medical School	Boston, MA, United States	Member	
Richard W	Issitt		DClinP	Great Ormond Street Hospital for Chi	London, United Kingdom	Member	
Anne Sophie	Jannot			HEGP, APHP Greater Paris Universit	Paris, France	Member	
Vianney	Jouhet		MD,PhD	Bordeaux University Hospital / ERIAS	Bordeaux, France	Member	
Ramakanth	Kavuluru		PhD	University of Kentucky	Lexington, KY, United State	Member	
Mark S	Keller			Harvard Medical School	Boston, MA, United States	Member	

Supplemental Online Content: Nonauthor Collaborators
*First name, last name, and suffix (if applicable) are required and will appear in PubMed.

***First Name and Middle Initial(s)**	***Last Name**	***Suffix (eg, Jr, III)**	Academic Degrees	Institution	Location (city, state/province, country)	Role or Contribution, eg, chair, principal investigator	Group (if more than 1 Group listed in the byline) and/or Subgroup (eg, Steering Committee)
Chris J	Kennedy		PhD	Massachusetts General Hospital	Boston, MA, United States	Member	
Kate F	Kernan		MD	Children's Hospital of PIttsburgh	Pittsburgh, PA, United Stat	Member	
Daniel A	Key		BEng	Great Ormond Street Hospital for Chi	London, United Kingdom	Member	
Katie	Kirchoff		MSHI	Medical University of South Carolina	Charleston, SC, United Stat	Member	
Jeffrey G	Klann		MEng, PhD	Massachusetts General Hospital	Boston, MA, United States	Member	
Isaac S	Kohane		MD, PhD	Harvard Medical School	Boston, MA, United States	Member	
Ian D	Krantz			The Children's Hospital of Philadelphi	Philadelphia, PA, United St	Member	
Detlef	Kraska		Dr.	University Hospital Erlangen	Erlangen, Germany	Member	
Ashok K	Krishnamurthy		PhD	University of North Carolina, Chapel H	Chapel Hill, NC, United Stat	Member	
Sehi	L'Yi		PhD	Harvard Medical School	Boston, MA, United States	Member	
Trang T	Le		PhD	University of Pennsylvania Perelman	Philadelphia, PA, United St	Member	
Judith	Leblanc			APHP Greater Paris University Hospi	Paris, France	Member	
Guillaume	Lemaitre			Université Paris-Saclay, Inria, CEA	Palaiseau, France	Member	
Leslie	Lenert		MD, MS	Medical University of South Carolina	Charleston, SC, United Stat	Member	
Damien	Leprovost			Clevy.io	Paris, France	Member	
Molei	Liu		PhD	Harvard T. H. Chan School of Public	Boston, MA, United States	Member	
Qi	Long		PhD	University of Pennsylvania Perelman	Philadelphia, PA, United St	Member	
Sara	Lozano-Zahonero		PhD	Faculty of Medicine and Medical Cen	Freiburg, Germany	Member	
Kristine E	Lynch		PhD	VA Salt Lake City Health Care Syster	Salt Lake City, UT, United S	Member	
Sadiqa	Mahmood			Health Catalyst, INC.	Cambridge, MA, United Sta	Member	
Sarah E	Maidlow		AA	University of Michigan	Ann Arbor, MI, United Stat	Member	
Adeline	Makoudjou		MD	Faculty of Medicine and Medical Cen	Freiburg, Germany	Member	
Alberto	Malovini		PhD	Istituti Clinici Scientifici Maugeri SpA	Pavia, Italy	Member	
Kenneth D	Mandl		MD, MPH	Boston Children's Hospital	Boston, MA, United States	Member	
Chengsheng	Mao		PhD	Northwestern University	Chicago, IL, United States	Member	
Anupama	Maram		MS	Harvard Medical School	Boston, MA, United States	Member	
Patricia	Martel			APHP Greater Paris University Hospi	Boulogne-Billancourt, Fran	Member	
Marcelo R	Martins		MSc	Clinics hospital of the Botucatu Medic	Botucatu, Brazil	Member	
Jayson S	Marwaha		MD	Beth Israel Deaconess Medical Cente	Boston, MA, United States	Member	
Aaron J	Masino		PhD	Children's Hospital of Philadelphia	Philadelphia, PA, United St	Member	
Maria	Mazzitelli		PhD	University Magna Graecia of Catanza	Catanzaro, Italy	Member	

Supplemental Online Content: Nonauthor Collaborators
*First name, last name, and suffix (if applicable) are required and will appear in PubMed.

***First Name and Middle Initial(s)**	***Last Name**	***Suffix (eg, Jr, III)**	Academic Degrees	Institution	Location (city, state/province, country)	Role or Contribution, eg, chair, principal investigator	Group (if more than 1 Group listed in the byline) and/or Subgroup (eg, Steering Committee)
Arthur	Mensch			ENS, PSL University	Paris, France	Member	
Marianna	Milano		PhD	University Magna Graecia of Catanza	Catanzaro, Italy	Member	
Marcos F	Minicucci		MD, PhD	Internal Medicine Department of Botu	Botucatu, Brazil	Member	
Jason H	Moore		PhD	Cedars-Sinai Medical Center	West Hollywood, CA, Unite	Member	
Cinta	Moraleda		MD,PhD	Hospital Universitario 12 de Octubre,	Madrid, Spain	Member	
Jeffrey S	Morris			University of Pennsylvania Perelman	Berwyn, PA, United States	Member	
Michele	Morris		BA	University of Pittsburgh	Pittsburgh, PA, United Stat	Member	
Karyn L	Moshal			Great Ormond Street Hospital for Chi	London, United Kingdom	Member	
Sajad	Mousavi		PhD	Harvard Medical School	Boston, MA, United States	Member	
Danielle L	Mowery		PhD	University of Pennsylvania Perelman	Philadelphia, PA, United St	Member	
Douglas A	Murad			David Geffen School of Medicine at U	Los Angeles, CA, United Sta	Member	
Shawn N	Murphy		MD, PhD	Massachusetts General Hospital	Boston, MA, United States	Member	
Thomas P	Naughton		BA	Harvard Medical School	Boston, MA, United States	Member	
Carlos Tadeu B	Neto			Clinical Research Unit of Botucatu M	Botucatu, Brazil	Member	
Antoine	Neuraz		MD, PhD	Hôpital Necker-Enfants Malade, Assis	Paris, France	Member	
Jane	Newburger		MD, MPH	Boston Children's Hospital, Harvard M	Boston, MA, United States	Member	
Kee Yuan	Ngiam		MBBS, FR(	National University Health System Si	Singapore, Singapore	Member	
James B	Norman			Harvard Medical School	Boston, MA, United States	Member	
Jihad	Obeid		MD, FAMIA	Medical University of South Carolina	Charleston, SC, United Stat	Member	
Marina P	Okoshi		PhD	Internal Medicine Department of Botu	Botucatu, Brazil	Member	
Karen L	Olson		PhD	Boston Children's Hospital and Harva	Boston, MA, United States	Member	
Nina	Orlova			APHP Greater Paris University Hospi	Paris, France	Member	
Brian D	Ostasiewski		BS	Wake Forest School of Medicine	Winston Salem, NC, United	Member	
Nathan P	Palmer		PhD	Harvard Medical School	Boston, MA, United States	Member	
Nicolas	Paris			APHP Greater Paris University Hospi	Paris, France	Member	
Lav P	Patel		MS	University Of Kansas Medical Center	Kansas City, KS, United Stat	Member	
Miguel	Pedrera-Jiménez		MS	Hospital Universitario 12 de Octubre,	Madrid, Spain	Member	
Ashley C	Pfaff		MD	Beth Israel Deaconess Medical Cente	Boston, MA, United States	Member	
Emily R	Pfaff		PhD	UNC Chapel Hill	Chapel Hill, NC, United Stat	Member	
Danielle	Pillion		MS	Harvard Medical School	Boston, MA, United States	Member	
Sara	Pizzimenti		MS	IRCCS Ca' Granda Ospedale Maggio	Milan, Italy	Member	

*First Name and Middle Initial(s)	*Last Name	*Suffix (eg, Jr, III)	Academic Degrees	Institution	Location (city, state/province, country)	Role or Contribution, eg, chair, principal investigator	Group (if more than 1 Group listed in the byline) and/or Subgroup (eg, Steering Committee)
Tanu	Priya		BS	Northwestern University Feinberg Sch	Chicago, IL, United States	Member	
Hans U	Prokosch			University of Erlangen-Nürnberg	Erlangen, Germany	Member	
Robson A	Prudente		PhD	Clinical Research Unit São Paulo Sta	Botucatu, Brazil	Member	
Andrea	Prunotto		PhD	Faculty of Medicine and Medical Cen	Freiburg, Germany	Member	
Víctor	Quirós-González		MS	Hospital Universitario 12 de Octubre,	Madrid, Spain	Member	
Rachel B	Ramoni			Department of Veterans Affairs	Washington, DC, D.C., Unit	Member	
Maryna	Raskin			Health Catalyst, INC.	Cambridge, MA, United Sta	Member	
Siegbert	Rieg		MD	Medical Center – University of Freibu	Freiburg, Germany	Member	
Gustavo	Roig-Domínguez		MS	Hospital Universitario 12 de Octubre,	Madrid, Spain	Member	
Pablo	Rojo		MD,PhD	Hospital Universitario 12 de Octubre,	Madrid, Spain	Member	
Paula	Rubio-Mayo		MS	Hospital Universitario 12 de Octubre,	Madrid, Spain	Member	
Paolo	Sacchi		MD	Fondazione I.R.C.C.S. Policlinico San	Pavia, Italy	Member	
Elisa	Salamanca			APHP Greater Paris University Hospi	Paris, France	Member	
Malarkodi Jebathilag	Samayamuthu		MD	University of Pittsburgh	Pittsburgh, PA, United Stat	Member	
Arnaud	Sandrin			APHP Greater Paris University Hospi	Paris, France	Member	
Nandhini	Santhanam		MSc	University Medicine Mannheim, Heide	Mannheim, Germany	Member	
Janaina CC	Santos		MS	Clinical Research Unit of Botucatu Me	Botucatu, Brazil	Member	
Fernando J	Sanz Vidorreta			David Geffen School of Medicine at U	Los Angeles, CA, United Sta	Member	
Maria	Savino		MS	Management Engineer, Direction	Pavia, Italy	Member	
Emily R	Schriver		MS	University of Pennsylvania Health Sys	Philadelphia, PA, United St	Member	
Petra	Schubert		MPH	VA Boston Healthcare System	Boston, MA, United States	Member	
Juergen	Schuettler			University Hospital Erlangen, FAU Erl	Erlangen, Germany	Member	
Luigia	Scudeller		MD, MSc	IRCCS Ca' Granda Ospedale Maggio	Milan, Italy	Member	
Neil J	Sebire		MD, FRCPa	Great Ormond Street Hospital for Chi	London, United Kingdom	Member	
Pablo	Serrano-Balazote		MD,MS	Hospital Universitario 12 de Octubre,	Madrid, Spain	Member	
Patricia	Serre			APHP Greater Paris University Hospi	Paris, France	Member	
Mohsin	Shah		MSc	Great Ormond Street Hospital for Chi	London, United Kingdom	Member	
Zahra	Shakeri Hossein Abad		PhD	University of Toronto	Toronto, Canada	Member	
Domenick	Silvio			University of Michigan	Ann Arbor, MI, United Stat	Member	
Piotr	Sliz			Boston Children's Hospital	Boston, MA, United States	Member	
Jiyeon	Son		MD	University of Pittsburgh Medical Cent	Pittsburgh, PA, United Stat	Member	

Supplemental Online Content: Nonauthor Collaborators

*First name, last name, and suffix (if applicable) are required and will appear in PubMed.

***First Name and Middle Initial(s)**	***Last Name**	***Suffix (eg, Jr, III)**	Academic Degrees	Institution	Location (city, state/province, country)	Role or Contribution, eg, chair, principal investigator	Group (if more than 1 Group listed in the byline) and/or Subgroup (eg, Steering Committee)
Charles	Sonday			St. Luke's University Health Network,	Bethlehem, PA, United Stat	Member	
Anastasia	Spiridou		PhD	Great Ormond Street Hospital for Chi	London, United Kingdom	Member	
Zachary H	Strasser		MD	Massachusetts General Hospital	Boston, MA, United States	Member	
Bryce WQ	Tan		MBBS	National University Hospital, Singapo	Singapore, Singapore	Member	
Byorn WL	Tan		MBBS	National University Hospital, Singapo	Singapore, Singapore	Member	
Suzana E	Tanni		PhD	Internal Medicine Department of Botu	Botucatu, Brazil	Member	
Ana I	Terriza-Torres		MS	Hospital Universitario 12 de Octubre,	Madrid, Spain	Member	
Valentina	Tibollo		MS	Istituti Clinici Scientifici Maugeri SpA	Pavia, Italy	Member	
Patric	Tippmann		MSc	Institute of Medical Biometry and Stat	Freiburg, Germany	Member	
Emma MS	Toh			National University of Singapore	Singapore, Singapore	Member	
Carlo	Torti		PhD	University Magna Graecia of Catanza	Catanzaro, Italy	Member	
Enrico M	Trecarichi		PhD	University Magna Graecia of Catanza	Catanzaro, Italy	Member	
Andrew K	Vallejos			Clinical & Translational Science Instit	Milwaukee, WI, United Sta	Member	
Gael	Varoquaux			Université Paris-Saclay, Inria, CEA, M	Palaiseau, France	Member	
Margaret E	Vella		MPH	Harvard Medical School	Boston, MA, United States	Member	
Jill-Jênn	Vie			SequeL, Inria Lille	Villeneuve-d'Ascq, France	Member	
Michele	Vitacca		MD, PhD	ICS S. Maugeri IRCCS Pavia Italy	Lumezzane (Bs), Italy	Member	
Kavishwar B	Wagholikar		MBBS, PhD	Massachusetts General Hospital	Boston, MA, United States	Member	
Lemuel R	Waitman			University of Missouri, Columbia. MO	Columbia, MO, United Stat	Member	
Xuan	Wang		PhD	Harvard Medical School	Boston, MA, United States	Member	
Demian	Wassermann			Université Paris-Saclay, Inria, CEA	Palaiseau, France	Member	
Griffin M	Weber		MD, PhD	Harvard Medical School	Boston, MA, United States	Member	
Martin	Wolkewitz		PhD	Institute of Medical Biometry and Sta	Freiburg, Germany	Member	
Scott	Wong			National University Hospital, Singapo	Singapore, Singapore	Member	
Xin	Xiong		MS	Harvard T.H. Chan School of Public H	Boston, MA, United States	Member	
Ye	Ye		BMED, MS	University of Pittsburgh	Pittsburgh, PA, United Stat	Member	
Nadir	Yehya		MD, MSCE	Children's Hospital of Philadelphia an	Philadelphia, PA, United St	Member	
William	Yuan		PhD	Harvard Medical School	Boston, MA, United States	Member	
Alberto	Zambelli			ASST Papa Giovanni XXIII, Bergamo	Bergamo, Italy	Member	
Harrison G	Zhang		HSDG	Harvard Medical School	Boston, MA, United States	Member	
Daniela	Zöller		PhD	Faculty of Medicine and Medical Cen	Freiburg, Germany	Member	

Supplemental Online Content: Nonauthor Collaborators
*First name, last name, and suffix (if applicable) are required and will appear in PubMed.

***First Name and Middle Initial(s)**	***Last Name**	***Suffix (eg, Jr, III)**	Academic Degrees	Institution	Location (city, state/province, country)	Role or Contribution, eg, chair, principal investigator	Group (if more than 1 Group listed in the byline) and/or Subgroup (eg, Steering Committee)
Valentina	Zuccaro		MD	Fondazione I.R.C.C.S. Policlinico Sar	Pavia, Italy	Member	
Chiara	Zucco		PhD	University Magna Graecia of Catanza	Catanzaro, Italy	Member	
