# Supplementary material for: Hospitalizations Associated With Mental Health Conditions Among Adolescents in the US and France During the COVID-19 Pandemic
Source: JAMA Netw Open. 2022 Dec 13;5(12):e2246548. doi: 10.1001/jamanetworkopen.2022.46548 (PMC9856226; doi:10.1001/jamanetworkopen.2022.46548)
Supplement: Supplement 3. — Data Sharing Statement [file jamanetwopen-e2246548-s003.pdf]

Data Sharing Statement

Gutiérrez-Sacristán. Hospitalizations Associated With Mental Health Conditions Among Adolescents in the US and France During the COVID-19 Pandemic. *JAMA Netw Open*. Published December 13, 2022.
doi:10.1001/jamanetworkopen.2022.46548

Data
Data available: No

Additional Information
Explanation for why data not available: Institutional Review Board from each hospital doesn't allow to share patient-level data outside the institution. The code was run locally at each site with meta-analysis centrally As described in the paper, we do share: Data Dictionary: https://github.com/covidclinical/Phase2.2.psy_peds_trends/tree/main/public-data
Code run at each site: https://github.com/covidclinical/Phase2.2.psy_peds_trends/blob/main/psy_peds_3.Rmd
Metanalysis:
https://github.com/covidclinical/Phase2.2.psy_peds_trends/blob/main/metanalysis_visualization/manuscript_Figures.Rmd
